# MHD Dissipative Williamson Nanofluid Flow with Chemical Reaction Due to a Slippery Elastic Sheet Which Was Contained within a Porous Medium

**DOI:** 10.3390/mi13111879

**Published:** 2022-10-31

**Authors:** Haifaa Alrihieli, Mounirah Areshi, Elham Alali, Ahmed M. Megahed

**Affiliations:** 1 Department of Mathematics, Faculty of Science, University of Tabuk, Tabuk 71491, Saudi Arabia; 2 Department of Mathematics, Faculty of Science, Benha University, Benha 13518, Egypt

**Keywords:** MHD, non-newtonian nanofluid, porous medium, viscous dissipation, chemical reaction, slip velocity, variable fluid properties

## Abstract

The reasons why the model of non-Newtonian nanofluids is more applicable than other models, particularly those that take the porous medium into account, are studied here. Thus, we looked at the heat and mass transfer features of a non-Newtonian Williamson nanofluid flow due to a stretched sheet under the impact of chemical reactions, slip velocity, viscous dissipation, and the magnetic field in this article. The main focus is on a situation in which the properties of Williamson nanofluid, such as viscosity and thermal conductivity, change with temperature. After utilizing the shooting technique, a numerical solution to the suggested problem is provided. As a result, several graphs have been drawn to highlight how various physical characteristics that arise in the problems affect velocity, temperature, and concentration profiles. It was discovered that the heat and mass transmission processes are affected by the viscous dissipation phenomena, the slip velocity assumption, and the magnetic field. Theoretical and numerical results show a high level of qualitative agreement.

## 1. Introduction

Nanofluids have gained a lot of attention in recent decades due to their claimed unique features and potential applications. When nanoparticles are present, the thermophysical and transport properties of the base fluid are usually altered. The term “nanofluid” refers to a colloidal dispersion of nanoparticles in a base fluid [1]. Rapid technological advancement necessitates the employment of a high-performing coolant in many thermal management activities. Nanofluids have crucial qualities that can not be overlooked when it comes to thermal regulation [2,3]. They also offer a significant potential for improving heat transfer rates in engineering systems, particularly for cooling electronic equipment [4,5,6,7]. The inclusion of nanoparticles in the base fluid, as many authors [8,9,10,11,12,13,14,15,16] have already said, has a considerable impact on the fluid’s physical characteristics and significantly increases the heat transmission mechanism. The Buongiorno nanofluid model [17], Williamson nanofluid model [18], micropolar model [19], and Casson-Williamson nanofluid model [20] have all been proposed to characterize the behaviour of nanofluids. It is also worth noting that MHD nanofluid flow research is crucial because of its essential industrial applications [21,22,23]. On the other hand, many research studies have concentrated on the chemical reaction phenomenon in nanofluid flow in conjunction with magnetic field influence [24], while others have focused on the chemical reaction phenomenon in a porous media [25] and its effect on nanofluid flow in a range of geometries.

The viscous dissipation phenomena is another essential aspect that should not be overlooked when studying nanofluid flow. Internal friction in a viscous flow causes an irreversible conversion of kinetic energy to thermal energy, which is known as the irreversible process of mechanical energy conversion to thermal energy. This important phenomenon only occurs if the fluid has a high viscosity and velocity [26,27,28]. The slip velocity is another important factor that influences the flow behavior of nanofluids. This phenomenon can occur in a variety of physical conditions, including internal cavity polishing, artificial heart valves, and micro electronics [29,30,31]. After reviewing the literature and being inspired by the prominent features of viscous dissipation, variable fluid properties, magnetic field, chemical reaction, and slip velocity phenomena, it was determined that non-Newtonian Williamson nanofluid flow in these significant physical situations has yet to be investigated. Due to the significance of this kind of fluid, the novelty of the current research is based on examining the problem of the flow and heat transmission mechanisms caused by a stretching sheet embedded in a porous medium while taking slip velocity and the impacts of chemical reactions into consideration.

## 2. Description of the Problem

**Figure 1:**
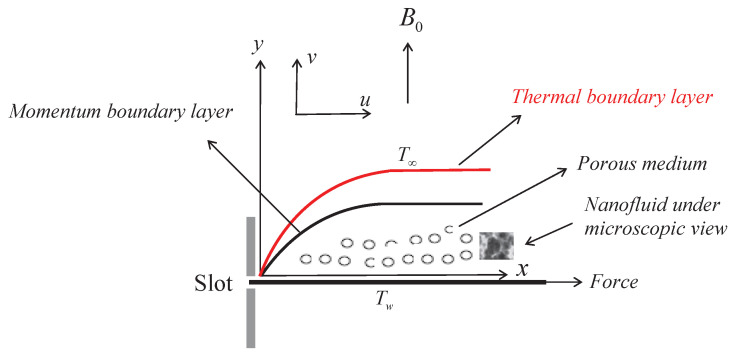
Physical model with coordinates.

A two-dimensional non-Newtonian Williamson nanofluid, which is characterized by the Williamson parameter Γ, will flow across an impermeable horizontal heated linearly stretched sheet immersed in a porous liquid in the current theoretical investigation. The nanoparticles in the nanofluid are assumed to circulate due to the Brownian diffusion coefficient $D_{B}$. In addition, the chemical conversion rate *K* is supposed to be applied to the nanofluid particles. The viscosity μ and thermal conductivity κ of the Williamson nanofluid are both assumed to be temperature functions in these formulae $\mu =\mu_{\infty }e^{-\alpha \theta }$ and $\kappa =\kappa_{\infty }\left(1+\varepsilon \theta \right)$ [32], but its density ρ is assumed to be constant. where $\mu_{\infty }$ is a constant viscosity of the nanofluid at the ambient, α is the viscosity parameter, $\kappa_{\infty }$ and ε are constant thermal conductivity and the thermal conductivity parameter conductivity, respectively. $T_{w}$ and $C_{w}$ are the temperature and concentration near the wall, while $T_{\infty }$ and $C_{\infty }$ are the temperature and concentration far away from the wall, as shown in Figure 1.

In addition, *T* denotes the temperature of the nanofluid, *C* the concentration of the nanofluid, and *u* and *v* the velocity components of the nanofluid. The problem is modelled using the following governing equations that follow the above-mentioned nanofluid flow conditions [33,34]:

(1) $$\frac{\partial u}{\partial x}+\frac{\partial v}{\partial y}=0,$$

(2) $$u\frac{\partial u}{\partial x}+v\frac{\partial u}{\partial y}=\frac{1}{\rho_{\infty }}\frac{\partial }{\partial y}\left\{\mu \frac{\partial u}{\partial y}+\mu \frac{\Gamma }{\sqrt{2}}{\left(\frac{\partial u}{\partial y}\right)}^{2}\right\}-\frac{\sigma B_{0}^{2}}{\rho_{\infty }}u-\frac{\mu }{\rho_{\infty }k}u,$$

(3) $$\begin{array}{cc} u\frac{\partial T}{\partial x}+v\frac{\partial T}{\partial y}=\frac{1}{\rho_{\infty }c_{p}}\frac{\partial }{\partial y}(\kappa \frac{\partial T}{\partial y}) & +\tau \left\{D_{B}\frac{\partial C}{\partial y}\frac{\partial T}{\partial y}+\frac{D_{T}}{T_{\infty }}{\left(\frac{\partial T}{\partial y}\right)}^{2}\right\}\\ & +\frac{\mu }{\rho_{\infty }c_{p}}\left[{\left(\frac{\partial u}{\partial y}\right)}^{2}+\frac{\Gamma }{\sqrt{2}}{\left(\frac{\partial u}{\partial y}\right)}^{3}\right], \end{array}$$

(4) $$u\frac{\partial C}{\partial x}+v\frac{\partial C}{\partial y}=D_{B}\frac{\partial^{2}C}{\partial y^{2}}-K\left(C-C_{\infty }\right)+\frac{D_{T}}{T_{\infty }}\frac{\partial^{2}T}{\partial y^{2}}.$$

As a result, the flow field is governed by the boundary conditions listed below [35]:

(5) $$u=ax+\frac{\lambda_{1}}{\mu_{\infty }}\left[\mu \frac{\partial u}{\partial y}+\mu \frac{\Gamma }{\sqrt{2}}{\left(\frac{\partial u}{\partial y}\right)}^{2}\right],\;v=0,\;T=T_{w},\;C=C_{w}\;\mathrm{at}\;y=0,$$

(6) $$u\to 0,\;T\to T_{\infty },\;C\to C_{\infty }\;\mathrm{as}\;y\to \infty ,$$

where *k* is the porous medium’s porosity, σ denotes the electrical conductivity of the nanofluid, $B_{0}$ is the magnetic field strength, $\lambda_{1}$ is the factor of the slip velocity and $D_{T}$ is the thermophoretic diffusion coefficient. Now, we begin the dimensionless quantities in the following forms before creating the solution algorithm:

(7) $$\eta ={\left(\frac{a}{\nu_{\infty }}\right)}^{\frac{1}{2}}y,\;u=axf^{\prime }\left(\eta \right),\;v=-{\left(a\nu_{\infty }\right)}^{\frac{1}{2}}f\left(\eta \right),$$

(8) $$\theta \left(\eta \right)=\frac{T-T_{\infty }}{T_{w}-T_{\infty }},\;\phi \left(\eta \right)=\frac{C-C_{\infty }}{C_{w}-C_{\infty }}.$$

Equation (1) becomes easily and quickly satisfied after applying these transformations (7) and (8), whereas Equations (2)–(4) with boundary conditions (5) and (6) become

(9) $$\left[\left(1+W_{e}f^{\prime \prime }\right)f^{\prime \prime \prime }-\alpha \theta^{\prime }f^{\prime \prime }\left(1+\frac{W_{e}}{2}f^{\prime \prime }\right)\right]e^{-\alpha \theta }+ff^{\prime \prime }-f^{\prime 2}-Mf^{\prime }-\delta e^{-\alpha \theta }f^{\prime }=0,$$

(10) $$\frac{1}{Pr}\left(\left(1+\varepsilon \theta \right)\theta^{\prime \prime }+\varepsilon \theta^{\prime 2}\right)+Nb\theta^{\prime }\phi^{\prime }+Nt{\left(\theta^{\prime }\right)}^{2}+f\theta^{\prime }+Ec\left[f^{\prime \prime 2}+\frac{W_{e}}{2}f^{\prime \prime 3}\right]e^{-\alpha \theta }=0,$$

(11) $$\phi^{\prime \prime }+Scf\phi^{\prime }-ScG\phi +\frac{Nt}{Nb}\theta^{\prime \prime }=0.$$

In addition, the boundary conditions become:

(12) $$f=0,\;f^{\prime }=1+\lambda \left[f^{\prime \prime }+\frac{W_{e}}{2}f^{\prime \prime 2}\right]e^{-\alpha \theta },\;\theta =1,\;\phi =1,\;at\;\eta =0,$$

(13) $$f^{\prime }\to 0,\;\theta \to 0,\;\phi \to 0,\;\mathrm{as}\;\;\eta \to \infty .$$

The velocity field, as well as the associated condition, is clearly regulated by the local Weissenberg number $W_{e}$, the magnetic parameter *M*, the porosity parameter δ and the slip velocity parameter λ, which respectively can be defined as:

(14) $$W_{e}=\Gamma x\sqrt{\frac{2a^{3}}{\nu_{\infty }}},M=\frac{\sigma \;B_{0}^{2}}{a\rho_{\infty }},\delta =\frac{\nu_{\infty }}{ka},\lambda =\lambda_{1}\sqrt{\frac{a}{\nu_{\infty }}}.$$

In addition, governing the temperature field are the Prandtl number Pr, the thermophoresis parameter Nt, the local Eckert number Ec, and the Brownian motion parameter Nb, which are defined as follows:

(15) $$Pr=\frac{\mu_{\infty }\;c_{p}}{\kappa_{\infty }},Nt=\frac{\tau D_{T}\left(T_{w}-T_{\infty }\right)}{T_{\infty }\nu_{\infty }},Ec=\frac{{\left(ax\right)}^{2}}{c_{p}\left(T_{w}-T_{\infty }\right)},Nb=\frac{\tau D_{B}\left(C_{w}-C_{\infty }\right)}{\nu_{\infty }}.$$

Finally, the concentration field is influenced by both basic factors, the chemical reaction parameter *G*, and the Schmidt number Sc, which have the following definitions:

(16) $$G=\frac{K}{a},Sc=\frac{\nu_{\infty }}{D_{B}}.$$

As we will see, in this case, the local parameters depending on the length scale *x* are the Williamson parameter $W_{e}$ and the Eckert number Ec. These parameters are a function of *x* and their changes occur locally throughout the flow action, hence it is important to note that the presented equation is only valid for a locally similar solution [36]. We have provided precise values of $W_{e}$ and Ec for the graphical findings, which relate to the flow study at a specific value of *x* and for all values of *y*. The following relations may now be achieved utilizing the above dimensionless transformations and well-defined relationships for wall shear stress Cf, local Nusselt number Nux, and local Sherwood number Shx:

(17) $$\begin{array}{cc} \; & C_{f}\sqrt{Re_{x}}=-\left[f^{\prime \prime }\left(0\right)+\frac{W_{e}}{2}f^{\prime \prime 2}\left(0\right)\right]e^{-\alpha \theta (0)},\\ & \frac{Nu_{x}}{\sqrt{Re_{x}}}=-\theta^{\prime }\left(0\right),\;\;\;\frac{Sh_{x}}{\sqrt{Re_{x}}}=-\phi^{\prime }\left(0\right), \end{array}$$

where $Re_{x}=\frac{u_{w}x}{\nu_{\infty }}$ is the local Reynolds number.

## 3. Results and Discussion

To provide a numerical solution for the suggested problem in this study, we used the shooting approach. The boundary value problem is reduced to identifying the initial conditions that produce a root by using the shooting technique. The shooting approach has the advantages of employing the speed and adaptability of approaches for initial value problems. Additionally, compared to other methods, this method significantly improves numerical stability and nonlinearity distribution. We set up a comparison table at the beginning of this section, which is shown in Table 1, to verify our computations, which were made using the shooting method, with previous results. Clearly, the findings are very similar to the previously published research of Khan and Pop [37], implying that the numerical method presented here is very suitable for this type of model.

**Figure 2:**
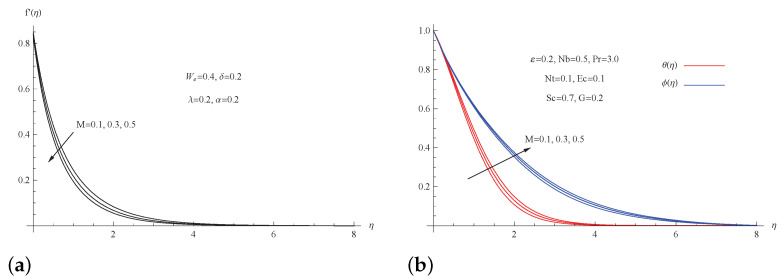
(**a**) $f^{\prime }\left(\eta \right)$ for picked *M*; (**b**) θ(η) and ϕ(η) for picked *M*.

The major goal of this part is to use graphical figures to illustrate the physics of the numerical non-Newtonian Williamson nanofluid model. The following figures show the impacts of various flow parameters on nanofluid flow behavior and heat mass characteristics, such as the magnetic parameter *M*, the porosity parameter δ, the slip velocity parameter λ, the thermophoresis parameter Nt, the Eckert number Ec, the Brownian motion parameter Nb, and the chemical reaction parameter *G*. Figure 2 shows how varying values of the magnetic parameter *M* affect velocity, temperature, and nanoparticle concentration. It has been demonstrated that boosting the magnetic field parameter *M* lessens both the momentum thickness and the velocity distribution, whereas the temperature and concentration distributions have the opposite effect. Physically, the magnetic field restricts the boundary layer with a resistive-type force. The only purpose of this force is to reduce the nanofluid’s speed. As a result, the nanofluid consequently absorbs additional heat energy from the same force.

**Figure 3:**
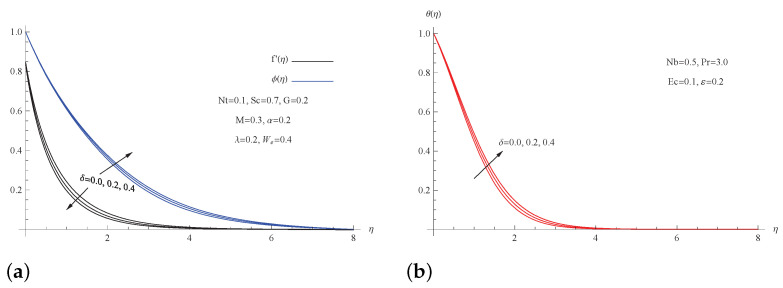
(**a**) $f^{\prime }\left(\eta \right)$ and ϕ(η) for picked δ; (**b**) θ(η) for picked δ.

Figure 3 depicts the effect of porosity parameter δ alteration on velocity, temperature, and nanoparticle concentration field. The figure indicates an enhanced tendency in both the temperature and concentration fields as the porosity parameter is increased, whereas the velocity field displays the opposite trend. Physically, increasing the porosity parameter leads to impeding the acceleration of the nanofluid flow, which in turn produces a reduction in the flow’s speed and an increase in the friction between the fluid particles, which raises the temperature field.

**Figure 4:**
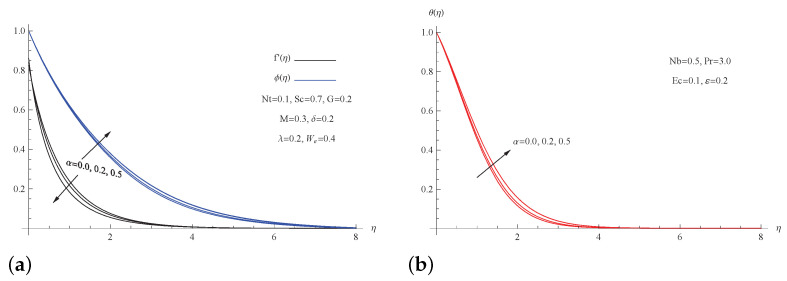
(**a**) $f^{\prime }\left(\eta \right)$ and ϕ(η) for picked α; (**b**) θ(η) for picked α.

Figure 4 shows the velocity of the nanofluid, its temperature, and the concentration of nanoparticles vs. various viscosity parameter α. As seen in this figure, a high value of the viscosity parameter α causes a decrease in the velocity field, whereas the same parameter generates an enhancement in both the temperature and concentration fields, resulting in an increase in the thermal boundary thickness. The pushing force diminishes as the nanofluid becomes more viscous, which results in a reduction in the nanofluid motion throughout the boundary layer, according to the physical explanation for this phenomenon.

**Figure 5:**
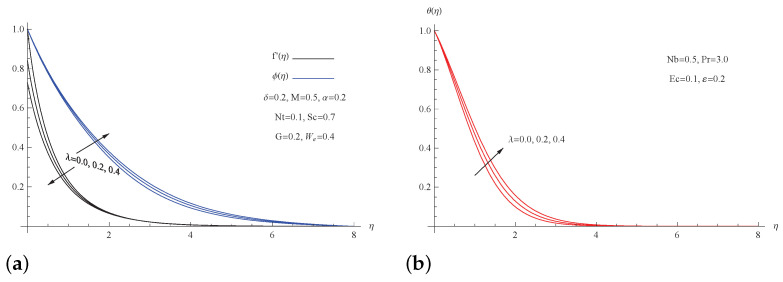
(**a**) $f^{\prime }\left(\eta \right)$ and ϕ(η) for picked λ; (**b**) θ(η) for picked λ.

The influence of the slip velocity parameter λ on the velocity $f^{\prime }\left(\eta \right)$, temperature θ(η), and nanoparticle concentration ϕ(η) profiles is shown in Figure 5. The temperature of the nanofluid θ(η) and the concentration profile ϕ(η) of nanoparticles both increase significantly as the slip velocity parameter λ is increased, whereas the velocity profile $f^{\prime }\left(\eta \right)$ is changed in the opposite direction.

**Figure 6:**
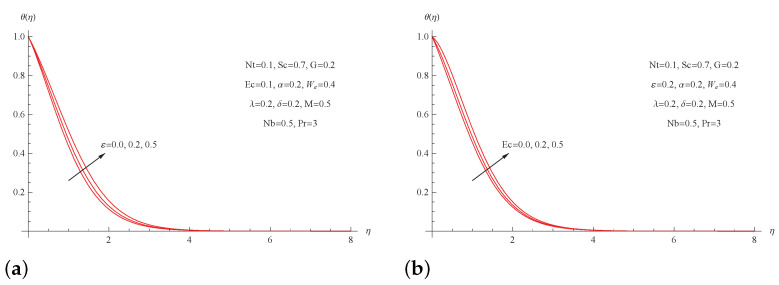
(**a**) θ(η) for picked ε; (**b**) θ(η) for picked Ec.

Figure 6 describes the results of the thermal conductivity parameter ε and the Eckert number Ec on the temperature profiles θ(η). Clearly, raising both the thermal conductivity parameter ε and the Eckert number Ec increases the thickness of the thermal boundary layer as well as the temperature profile of the nanofluid. In terms of physics, the existence of a viscous dissipation phenomena or the dependence of the conductivity of nanofluids on temperature has the result of raising the fluid temperature, which enhances the thickness of the thermal boundary layer.

**Figure 7:**
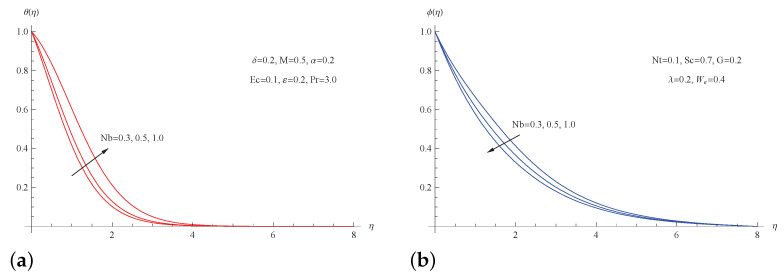
(**a**) θ(η) for picked *G*; (**b**) ϕ(η) for picked *G*.

The influence of the Brownian motion parameter Nb on temperature and concentration is depicted in Figure 7. The thermal thickness as well as the temperature distribution θ(η) grow rapidly as Nb increases, whereas the nanoparticle concentration distribution ϕ(η) goes in the other direction with the same parameter. Physically, the Brownian motion parameter depends on the Brownian diffusion coefficient, and an increase in the Brownian motion parameter is associated with an increase in the heat diffusion and the temperature profile.

Table 2 summarizes the findings of the skin friction coefficient $C_{f}\sqrt{Re_{x}}$, wall temperature gradient $\frac{Nu_{x}}{\sqrt{Re_{x}}}$, and wall concentration gradient $\frac{Sh_{x}}{\sqrt{Re_{x}}}$ as a function of the parameters regulating the boundary layer region studied here. Table 2 shows that the local skin friction coefficient increases as the viscosity parameter, magnetic number, and porosity parameters increase, whereas the local Nusselt number and local Sherwood number for the same parameters show the opposite tendency. As the slip velocity parameter is maintained, the local skin-friction coefficient, surface heat transfer rate, and surface mass transfer rate all reduce. It is due to the fact that, as both the thermal conductivity parameter and the chemical reaction parameter expand, the surface mass transfer rate increases, whilst the local skin-friction coefficient and the local Nusselt number decrease. Additionally, increases in the Eckert number or Brownian motion parameter diminish both the rate of heat transmission and the value of the skin friction coefficient, but the local Sherwood number has the inverse result.

## 4. Conclusions

In this research, we looked at the heat and mass transfer mechanisms for non-Newtonian Williamson nanofluid flow caused by an elastic sheet with chemical reaction, slip velocity, and viscous dissipation. The sheet is assumed to be stretched linearly, exposed to a magnetic field, and submerged in a saturated porous medium. Using a shooting approach, the governing boundary layer equations are numerically solved for several values of the associated parameters. The following findings can be taken from the research work:

1. The viscosity parameter, the porous parameter, and the magnetic parameter all show a decrease in velocity.
2. Temperature rises as the Eckert number, magnetic parameter, porosity parameter, and slip velocity parameter increase.
3. When the viscosity parameter, magnetic number, and porous parameter are increased, the skin-friction coefficient rises, whereas the chemical reaction parameter, Brownian motion parameter, and Eckert number grow in the opposite direction.
4. The skin-friction coefficient and the local Nusselt number decline for the mounting values of thermal conductivity parameter and the Eckert number, whereas an enhancement in the local Sherwood number is observed.
5. When the Brownian motion parameter and the chemical reaction parameter are both increased, the local Sherwood number increases; however, the increasing values of the slip velocity parameter and viscosity parameter have the opposite effect.

## Figures and Tables

**Table 1:** Comparison of $-\theta^{\prime }\left(0\right)$ with the results of Khan and Pop [37] when $\alpha =M=\delta =\varepsilon =Ec=\lambda =W_{e}=0$ and Pr=10.

Nb	Nt	Khan and Pop [37]	Present Work
0.1	0.1	0.9524	0.95239854
0.2	0.2	0.3654	0.36540037
0.3	0.3	0.1355	0.13548752
0.4	0.4	0.0495	0.04949979
0.5	0.5	0.0179	0.01787205

**Table 2:** Values of $C_{f}\sqrt{Re_{x}}$, $\frac{Nu_{x}}{\sqrt{Re_{x}}}$ and $\frac{Sh_{x}}{\sqrt{Re_{x}}}$ for various values of M,δ,α,λ,ε,Nb,Ec and *G* with $Nt=0.1,W_{e}=0.4,Pr=3.0$ and Sc=0.7.

*M*	δ	α	λ	ε	Ec	Nb	*G*	CfRex	NuxRex	ShxRex
0.1	0.2	0.2	0.2	0.2	0.1	0.5	0.2	0.924495	0.442366	0.503462
0.3	0.2	0.2	0.2	0.2	0.1	0.5	0.2	0.980413	0.416318	0.495631
0.5	0.2	0.2	0.2	0.2	0.1	0.5	0.2	1.030441	0.392609	0.489825
0.3	0.0	0.2	0.2	0.2	0.1	0.5	0.2	0.932834	0.439496	0.502721
0.3	0.2	0.2	0.2	0.2	0.1	0.5	0.2	0.980413	0.416318	0.495631
0.3	0.4	0.2	0.2	0.2	0.1	0.5	0.2	1.023210	0.395206	0.490356
0.3	0.2	0.0	0.2	0.2	0.1	0.5	0.2	0.874810	0.428961	0.501554
0.3	0.2	0.2	0.2	0.2	0.1	0.5	0.2	0.980413	0.416318	0.495631
0.3	0.2	0.5	0.2	0.2	0.1	0.5	0.2	1.147720	0.394267	0.486333
0.3	0.2	0.2	0.0	0.2	0.1	0.5	0.2	1.188541	0.434861	0.514142
0.3	0.2	0.2	0.2	0.2	0.1	0.5	0.2	0.980413	0.416318	0.495631
0.3	0.2	0.2	0.4	0.2	0.1	0.5	0.2	0.830424	0.394087	0.482866
0.3	0.2	0.2	0.2	0.0	0.1	0.5	0.2	0.981487	0.450101	0.490738
0.3	0.2	0.2	0.2	0.2	0.1	0.5	0.2	0.980413	0.416318	0.495631
0.3	0.2	0.2	0.2	0.5	0.1	0.5	0.2	0.979121	0.377751	0.501609
0.3	0.2	0.2	0.2	0.2	0.0	0.5	0.2	0.981267	0.482538	0.483647
0.3	0.2	0.2	0.2	0.2	0.2	0.5	0.2	0.979578	0.350767	0.508082
0.3	0.2	0.2	0.2	0.2	0.5	0.5	0.2	0.977066	0.153552	0.544668
0.3	0.2	0.2	0.2	0.2	0.1	0.3	0.2	0.982558	0.524028	0.441149
0.3	0.2	0.2	0.2	0.2	0.1	0.5	0.2	0.980413	0.416318	0.495631
0.3	0.2	0.2	0.2	0.2	0.1	1.0	0.2	0.975782	0.212324	0.530527
0.3	0.2	0.2	0.2	0.2	0.1	0.5	0.0	0.981627	0.487031	0.278046
0.3	0.2	0.2	0.2	0.2	0.1	0.5	0.2	0.980413	0.416318	0.495631
0.3	0.2	0.2	0.2	0.2	0.1	0.5	0.5	0.979654	0.368335	0.703169

## Data Availability

Not applicable.
